# Vitamin D deficiency and supplementation in critical illness—the known knowns and known unknowns

**DOI:** 10.1186/s13054-018-2185-8

**Published:** 2018-10-29

**Authors:** Priya Nair, Balasubramaniam Venkatesh, Jacqueline R Center

**Affiliations:** 1 St Vincents Hospital, Sydney, Australia; 2 University of New South Wales, Sydney, Australia; 3 Garvan Institute for Medical Research, Sydney, Australia; 4 George Institute for Global Health, Sydney, Australia; 5 Wesley Hospital, Brisbane, Australia; 6 Princess Alexandra Hospital, Brisbane, Australia; 7 University of Queensland, Brisbane, Australia; 8 Intensive Care Unit, St Vincents Hospital, Victoria Street, Darlinghurst, NSW 2010 Australia

**Keywords:** Vitamin D, Pleiotropic effects, Cholecalciferol, Supplementation, Vitamin D binding protein

## Abstract

The burgeoning literature on vitamin D deficiency and supplementation over the past decade or so has generated a greater understanding of some areas but also an appreciation of the many areas of equipoise. This is particularly relevant in the field of critical care with the heterogeneous patient populations, the severity and duration of illness and the frequency of comorbid conditions.

This review aims to summarise the current knowledge base of vitamin D deficiency within the context of critical illness—“the known knowns”—and also highlight the areas of recognised uncertainty—“the known unknowns”. It acknowledges the fact that there may well be other knowledge gaps of clinical relevance of which we are currently unaware—“the unknown unknowns”.

## The known knowns

### Pleiotropic functions of vitamin D

Vitamin D_3_ is produced in the skin from 7-dehydrocholesterol in a dual-stage process where the B ring is broken under ultraviolet rays (e.g. sunlight), and the pre-D_3_ formed in this process isomerises to D_3_ in a thermo-sensitive but non-catalytic process [1].

The three main steps in vitamin D metabolism, 25-hydroxylation, 1a-hydroxylation, and 24-hydroxylation, are all performed by cytochrome P450 mixed-function oxidases (CYPs). The first step towards activation is conversion of vitamin D to 25 hydroxy-D. In addition to UV activation small amounts of vitamin D, either as D2 or D3, can enter the body from its intestinal absorption from dietary intake and progress towards activation by hydroxlyation. The next step toward full activation is into 1,25 dihydroxy-vitamin D (1,25(OH)_2_D) via CYP27B1 (also known as 1-alpha hydroxylase), a mitochondrial P450 enzyme in the proximal renal tubule of the kidney. 25-Hydroxyvitamin D 24 hydroxylase, also known as CYP24, can hydroxylate both 25 hydroxy-D and 1,25(OH)_2_D. In addition to 1,25(OH)_2_D, the kidney also produces 24,25 dihydroxyvitamin D, a relatively inactive metabolite [2].

**Fig. 1:**
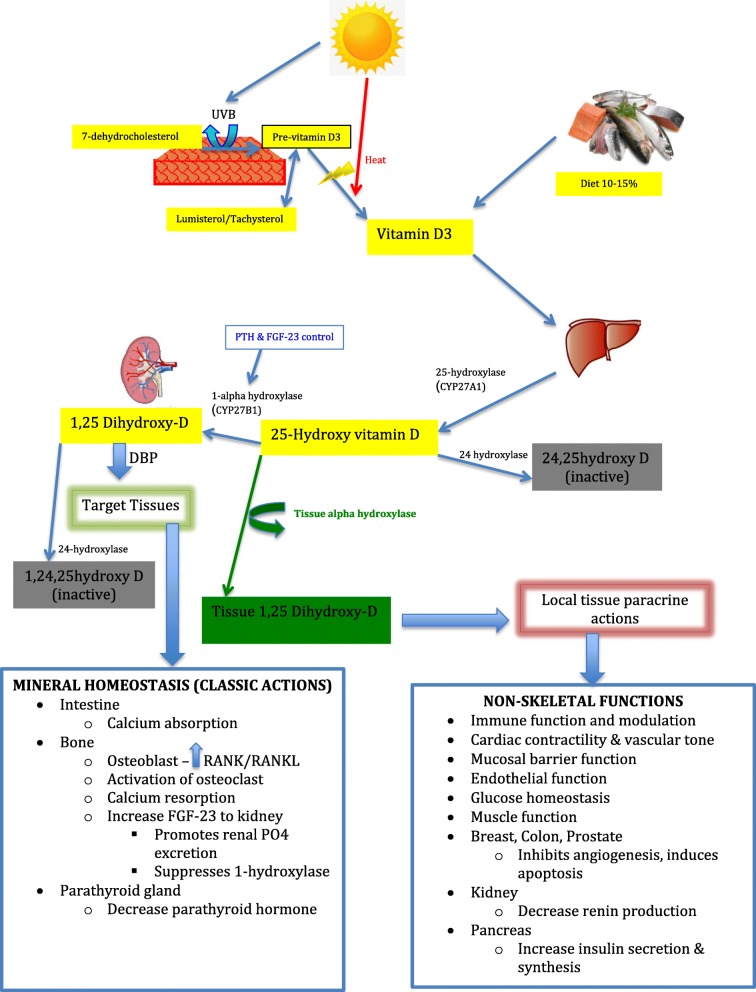
Synthesis, metabolism and functions of vitamin D. *PTH* parathyroid hormone, *FGF-23* fibroblast growth factor-23, *RANKL* receptor activator of nuclear kappa-B ligand

Figure 1 summarises the synthesis and metabolism of vitamin D as well as its classic and pleiotropic functions.

There is a growing appreciation for the many roles of vitamin D beyond its classic actions on calcium metabolism and musculoskeletal health. This has been driven by the finding that most body tissues have receptors for the active form of vitamin D, 1,25(OH)_2_D, known as vitamin D receptors (VDRs). Additionally, most of these tissues also contain the enzyme CYP27B1, which is responsible for the conversion of the major circulating form of vitamin D, 25-hydroxy-vitamin D (25 hydroxy-D), to its active metabolite 1,25(OH)_2_D. Regulation of this conversion at the tissue level differs from the conventional activation that occurs in the kidney in that it is more substrate dependent and hence more susceptible to vitamin D deficiency [2].

The non-skeletal actions of vitamin D are mediated by the control of gene expression in a number of organs such as the brain, prostate, colon and immune cells, which may be of particular relevance in critical illness. These non-skeletal actions result in regulation of cellular proliferation, differentiation, apoptosis and angiogenesis [3]. In fact the mechanism of action of vitamin D in these contexts is analogous to the way in which steroid hormones act. As a result of this contemporary knowledge, vitamin D is considered more a hormone than a vitamin [4].

1,25(OH)_2_D has been reported in animal models and in cultured cells to improve insulin production, modulate T-and B-cell activity, enhance phagocytic killing activity, improve vascular smooth muscle resistance, reduce risk of developing autoimmune diseases and inhibit cancer cell growth [5].

### Prevalence of deficiency with season and latitude

Vitamin D deficiency is highly prevalent in all age groups. Depending on the definition used, approximately 1 billion adults (15% of the population) are vitamin D deficient. Prevalence is higher in Middle Eastern countries where sun exposure is limited by clothing, especially in girls and women.

This high prevalence may be related to several factors, such as decreased vitamin D photosynthesis in response to UVB in individuals with high skin melanin content, aging, use of extensive skin coverage and minimal exposure to sunlight. In addition, low vitamin D intake and high rates of obesity contribute [6].

Season appears to be a small part of the problem worldwide, as countries with long winters appear to have lower deficiency rates overall compared to sunny countries, which is probably related to the fortification of staples, consumption of fatty fish and regular use of vitamin D supplements [6]. However, seasonal variation in vitamin D has been well documented with 25 hydroxy-D levels varying by 10–20 nmol/L between summer and winter [7].

Although it has been hypothesised that influenza pandemics are associated with solar control of vitamin D levels in humans, which waxes and wanes along with solar cycle-dependent ultraviolet radiation [8], other groups have refuted this [9].

In a cohort study of critically ill adults in France, admission to the ICU in spring (following winter months) was found to be an independent predictor of severe vitamin D deficiency (level < 30 nmol/L) [10].

### Association of deficiency with adverse health outcomes

A number of population studies have shown low vitamin D levels are associated with poor outcomes. However, causality is more difficult to establish given that a low vitamin D level in itself might be a marker of poor general health [11], with deficiency observed in individuals with limited physical activity and sunlight exposure, advanced age, obesity, poor diet and other comorbid illnesses.

In the general population, mortality risk appears to decrease as 25 hydroxy-D levels increase, with optimal levels 75–87.5 nmol/l. A large meta-analysis of community-dwelling adults showed that the lowest 25 hydroxy-D quintile observed was associated with increased all-cause mortality (pooled risk ratio of 1.57; 95% CI 1.36 to 1.81) [12].

Conditions that have been associated with vitamin D deficiency include certain malignancies such as colon, breast, ovarian, prostate and lymphoma. Some studies also report increased mortality risk with these cancers in vitamin D-deficient individuals [13–16]. Similarly low vitamin D levels have been associated with cardiovascular conditions such as poor hypertension control and congestive cardiac failure [17, 18]. Vitamin D deficient subjects who have multiple sclerosis, diabetes, depression and certain infections such as influenza, tuberculosis and other conditions have demonstrated similar association with adverse outcomes [19–23].

### Prevalence of deficiency in critical illness and association with poor outcomes

Several observational studies in critically ill patients have demonstrated an association between vitamin D deficiency/insufficiency and adverse outcomes [10, 24–32]. These include higher illness severity scores and risk of death, longer stay in ICU, longer duration of mechanical ventilation, increased rates of ventilator-associated pneumonia, blood culture positivity and an increased incidence of organ dysfunction, particularly acute kidney injury. The associated ICU and hospital costs are also higher in vitamin D-deficient patients [33].

A systematic review of 14 observational studies involving 9,715 critically ill patients reported an increased association with sepsis and increased risk of death and concluded that vitamin D deficiency was associated with an increased susceptibility for severe infections and risk of death in critically ill patients [34]. This suggests that vitamin D deficiency might serve as a predictor of these negative outcomes in the ICU.

Likewise in the paediatric intensive care unit (PICU) population, a recent systematic review and meta-analysis has reported a 50% prevalence of deficiency at the time of PICU admission. Deficiency was also determined to be associated with greater illness severity, multiple organ dysfunction, and mortality in the PICU setting [35].

### Supplementation of vitamin D in critically ill patients is safe

A handful of studies have investigated vitamin D supplementation in ICU patients [36–42]. These studies have predominantly used oral cholecalciferol in doses varying from 200 to 540,000 IU in either single or repeated doses. None of the studies reported any clinically relevant adverse effects. Transient hypercalcaemia not requiring any intervention was the only reported finding [42].

In a randomised controlled trial (RCT) by Amrein et al. [40], mild hypercalcaemia was the major adverse effect associated with high-dose vitamin D, but no serious adverse events were recorded. Mean calcium and phosphorus levels were similar between the placebo and vitamin D3 group. Serum ionised calcium levels were slightly higher in the vitamin D3 group only at the 6-month follow-up. The two highest 25 hydroxy-D levels recorded were below those considered to be acutely toxic (> 150 ng/mL). Individual hypercalcaemia did occur in some instances in the vitamin D3 group, but remained asymptomatic and did not require treatment. Renal parameters or the degree of hypercalciuria were not different between the groups.

This suggests that supplementation of vitamin D within these dose ranges is safe in the short term (up to 6 months) in critically ill patients, although none of the studies have published follow-up beyond that time period.

## The known unknowns

### The optimum range for vitamin D and what level to aim for?

In 2010, the Institute of Medicine (IOM) issued a report based on examination of data by a group of experts. They estimated that a vitamin D level of 50 nmol/L or higher was adequate for good bone health, and a level < 50 was considered a vitamin D deficiency [43].

In 2011, the Endocrine Society issued a report urging a much higher minimum blood level of vitamin D. The society’s clinical practice guideline was developed by experts in the field assigned to a Vitamin D Task Force, and based on all the evidence they recommended a minimum vitamin D level of 75 nmol/L and, because of the vagaries of some of the assays, levels between 100 and 150 nmol/L to guarantee sufficiency for both children and adults [44]. These reference ranges are determined by levels of vitamin D that achieve maximal parathyroid hormone suppression. However, this method for identifying the optimal level of vitamin D remains controversial; the relationship between serum 25 hydroxy-D and parathyroid hormone is inconsistent, and no clear threshold defining “sufficiency” has been established.

These recommendations from the Endocrine Society pertain to patients at risk of complications related to vitamin D deficiency, while those from the IOM on the other hand relate to healthy individuals.

Interestingly, although low levels of vitamin D have been associated with higher mortality and poor health outcomes, high levels may not be beneficial and could even be harmful. The first reports of a U-shaped or reverse J-shaped relationship were for prostate cancer [45]. Subsequently, this has been shown in other groups including with all-cause mortality [46].

Importantly, based on level of risk there is unlikely to be one single optimum level. For patients at high risk of poor bone health or colorectal cancer, accepting the potential risks of higher 25 hydroxy-D levels may be warranted to gain maximum possible benefits [47]. In addition to variation in the optimum level between individuals, the optimum level for different cellular functions and target actions within the same individual may be different; for example the bone health target may differ from that required for immune function.

Lastly, we currently have no data on optimum levels for critically ill patients. A study in intensive care patients demonstrated that supraphysiological levels of 25 hydroxy-D on ICU admission, akin to low levels, were associated with higher illness severity and mortality [48]. Another retrospective study of pre-admission 25 hydroxy-D levels in 24,094 adult patients showed that 25 hydroxy-D levels both less than 50 nmol/L and equal to or greater than 150 nmol/L before hospitalization were associated with increased odds of 90-day mortality [49].

### Which assay to use?

Challenges in regards to 25 hydroxy-D assays relate to several factors: it is hydrophobic and therefore unstable in water; its lipophilic nature means it strongly associates with vitamin D binding protein (DBP); endogenous lipids co-extract from plasma and serum, affecting binding and chromatographic separation; it exists in several molecular forms; direct natural sunlight degrades both 25 hydroxy-D and its metabolites.

Most commercial pathology laboratories use an automated immunoassay, such as the Diasorin Liaison, to measure the 25 hydroxy-D concentration. More specialised laboratories may use a liquid chromatography tandem mass spectrometry (LC-MS/MS) assay, which provides superior performance but is less amenable to high throughput [50]. An Australian study that examined agreement in 25 hydroxy-D concentrations measured by different assays and laboratories demonstrated that bias and variability in measurements sufficient to affect clinical decision-making were found both between laboratories and between assays with up to one in three subjects being misclassified as deficient depending on the laboratory and assay. For example serum 25 hydroxy-D concentrations of different aliquots of the same blood sample measured using Liaison at one laboratory were, on average, 26 nmol/L lower than those measured using LC-MS/MS (95% limits of agreement − 13.21, 65.31). The magnitude and variation in these differences increased at higher concentrations. This resulted in considerable misclassification of subjects into deficient or not, according to commonly used cut-points, with the frequency of misclassification slightly greater for a cut-point of 75 nmol/L compared with 50 nmol/L. [51]. Importantly, this lack of standardisation between laboratories and assays could affect the interpretation of the studies and meta-analyses on vitamin D deficiency discussed in previous sections*.*

The recent introduction of international Standard Reference Materials (SRM), human serum-based SRMs since 2012, and the development and acceptance of reference method procedures allied to the technical innovations in sample processing and analysis should all contribute to an improvement in the accuracy, precision and harmonization of results generated by all methods in use currently and those developed in the future [52].

### Which metabolite to measure?

Over 40 metabolites of vitamin D have been identified, and this could potentially result in difficulties in establishing assays. In practice, however, the vast majority have a very short half-life and are thus of minimal interest. 25 Hydroxy-D has a half-life of 21–30 days and its measurement is therefore a better indicator of vitamin D stores, whether obtained from sunlight (ultraviolet exposure) or dietary sources. The most potent physiologically active circulating metabolite produced by humans is 1,25(OH)_2_D, which has a half-life of 4–15 h and while 25 hydroxy-D circulates in nanomole per litre concentrations, 1,25(OH)_2_D is present in picomole per litre concentrations, which means that it represents the greater challenge for accurate measurement.

Although the focus so far in most studies has been on the major storage form, 25 hydroxy-D, this may be an oversimplification as a change in 25 hydroxy-D concentration may represent far more than simply a shift in storage. Instead it could underpin physiologic and/or pathophysiologic responses in vitamin D transport (bound to DBP), activation (to 1,25(OH)_2_D) and/or catabolism (to 24,25(OH)_2_D). This may be of particular relevance in the understanding of the extra-skeletal actions of vitamin D. In addition, particularly in critical illness with associated organ dysfunction, hepatic and renal activation may be affected.

More recently, it has been hypothesized that metabolic profiles may differ between critically ill patients relative to their vitamin D status. Lasky-Su et al. [53] found that in patients with the systemic inflammatory response or sepsis, metabolic profiles differed significantly in patients with 25 hydroxy-D levels < 37.5nnmol/L compared with those with levels > 37.5 nmol/L. They found that the glutathione and glutamate pathways particularly were altered with vitamin D status. These pathways play a role in oxidation and immunomodulation, respectively. This is therefore another potential area of interest when assessing vitamin D status in critical illness.

### Should we measure free vitamin D levels—the role of DBP

As alluded to above, the majority of circulating 25 hydroxy-D and 1,25(OH)_2_D is tightly bound to DBP and albumin, with less than 1% circulating in an unbound form. Binding of DBP impairs delivery of 25 hydroxy-D to vitamin D-activating 1-alpha-hydroxylase in target cells. It is therefore the unbound or free form of these metabolites which is active in most cells. As a result, factors affecting DBP alter the interpretation of 25 hydroxy-D levels [54]. Levels of DBP are affected by genetic factors, certain drugs (tenofovir, aspirin, oral contraceptive pill), smoking, hormonal factors, obesity and insulin resistance, end stage liver disease and nephrotic syndrome [54].

It might, therefore, be important clinically to be able to measure the free and bound fractions by using a DBP assay. However, measurement of DBP is not standardized and, as a result, it is difficult to compare the different assays and studies.

Alternatively, free 25 hydroxy-D levels can be directly measured by centrifugal ultrafiltration and a newer commercially available enzyme-linked immunosorbent assay. Centrifugal ultrafiltration is the gold standard but not commonly utilized as it is technically difficult, expensive and requires a very sensitive assay methodology [55].

Gene sequencing has uncovered many variations in the DBP gene. Over 120 variants of DBP have been found and, of these, three main phenotypic variants have been described. The variants have different characteristics that can alter 25 hydroxy-D levels. Powe et al.[56] demonstrated in a randomized, placebo-controlled trial that variations in race altered DBP levels and no change in DBP occurred with replacement of vitamin D. In addition, black subjects were found to have lower DBP levels than non-black subjects, resulting in similar concentrations of estimated bioavailable 25 hydroxy-D.

In critically ill patients, DBP falls during the systemic inflammatory response, which theoretically is related to a fall in 25 hydroxy-D. This was discovered in a study performed on patients undergoing orthopaedic surgery who were found to have elevated acute phase reactants [57].

A study performed on ICU patients showed that bactericidal activity was associated with 25 hydroxy-D concentrations, and DBP was decreased in patients with sepsis in comparison to subjects without sepsis [58]. A paediatric intensive care study found that levels of DBP and total 25 hydroxy-D were lower than those reported in healthy children. The lower DBP levels increased bioavailability of 25 hydroxy-D but the calculated bioavailable 25 hydroxy-D levels were also inversely associated with illness severity [59].

Therefore, total 25 hydroxy-D might not be a reliable indicator in the critical care situation. Overall, assessing vitamin D status and its activity in ICU patients, the implication of free or bound vitamin all contribute to the complexity of designing trials in this area.

On the other hand, Martucci et al. [60] undertook a post-hoc analysis of the VITdAL-ICU trial patients where DBP was measured in stored samples. They found that high dose cholecalciferol increased both total and bioavailable vitamin D metabolites in a similar way. Calculating free levels, however, did not enhance the ability to predict mortality when compared to 25 hydroxy-D alone, suggesting that there may not be an additional benefit to analyzing free vitamin D levels.

### Can we rely on a single level of 25 hydroxy-D?

A single 25 hydroxy-D measurement provides a snapshot of current vitamin D status. This typically varies during the year, paralleling changes in the amount of sun exposure. In an ambulatory population, it is probably the best indicator of vitamin D status given its relatively long half-life and relatively high concentrations in the order of nanomoles per litre. However, in critically ill patients this might not be the case. A study that undertook hourly assays in a cohort of ICU patients demonstrated marked variability during a 24- h period [61]. The authors postulated alterations in DBP concentrations, fluid shifts and assay variability as potential explanations for these findings. Another consideration in critical illness is haemodilution during the acute resuscitation phase. A study using cardiopulmonary bypass as a model for haemodilution demonstrated that haemodilution significantly lowered serum 25 hydroxy-D levels, which took up to 24 h to resolve [62]. Therefore, interpreting a single measurement in critically ill patients to assess deficiency risk or consider supplementation requires caution.

### Is vitamin D deficiency a marker of severity of critical illness or does it contribute to poor outcomes?

As discussed above, vitamin D deficiency in critical illness has been associated with poor outcomes and increased mortality. Although there are biologically plausible mechanisms by which deficiency might contribute to these outcomes, such as immune dysfunction, cardiovascular disease, dysglycaemia, and endothelial and mucosal barrier disruption [63], no studies prove a causative link. It is possible, therefore, that the low levels observed are merely a marker of poor general health resulting in limited exposure to sunlight, chronic illness and poor diet and, therefore, associated with adverse outcomes with vitamin D deficiency being the innocent bystander to this inevitable trajectory.

### Should vitamin D supplementation be practiced widely?

Multiple RCTs on vitamin D supplementation in the general population have been conducted, with conflicting results. These trials have involved different subgroups and looked for different outcomes with variable baseline levels of vitamin D and supplementation doses.

In cancer patients, the greatest benefit has been seen when patients with levels < 50 nmol/L are randomised to receive supplementation. However, there also appears to be benefit in increasing levels from 75 to 100 nmol/L [64–66].

As an example [66], cancer incidence was lower in women supplemented with calcium and vitamin D compared to placebo control subjects (*P* < 0.03). With the use of logistic regression, the unadjusted relative risks (RR) of cancer incidence in the Calcium + D and Calcium-only groups were 0.402 (*p* = 0.01) and 0.532 (*P* = 0.06), respectively.

For cardiovascular and metabolic diseases, most RCTs do not find beneficial effects of vitamin D supplementation, even when baseline vitamin D levels are low [67]. However, there does seem to be some benefit for congestive heart failure. In these patients, after vitamin D supplementation, the serum level of pro-brain natriuretic peptide markedly decreased (*p* < 0.001). Restoration of serum 25 hydroxy-D level was also associated with substantial improvement in NYHA class (*p* < 0.001) and 6-min walk distance (*p* < 0.001) [68].

RCTs have found beneficial effects of vitamin D supplementation in influenza and acute respiratory tract infections (RTI) [7, 69, 70]. This includes a meta-analysis of randomized trials of vitamin D to prevent RTI. Overall, vitamin D showed a protective effect against RTI (OR 0.64; 95% CI 0.49 to 0.84). There was significant heterogeneity between studies. The protective effect was larger in studies using once-daily dosing compared to bolus doses (OR = 0.51 vs OR = 0.86, *p* = 0.01) [69].

A Cochrane meta-analysis of patients (paediatric and adult) with predominantly mild to moderate asthma suggests that vitamin D is likely to reduce the risk of severe asthma exacerbation and reduce health care use [71].

In a meta-analysis of RCTs assessing all-cause mortality rates from vitamin D supplementation, relative risks for all-cause mortality were 0.89 (0.80 to 0.99) for vitamin D3 supplementation and 1.04 (0.97 to 1.11) for vitamin D2 supplementation [72].

On the other hand a recent RCT reported an increased risk of falls with high-dose vitamin D supplementation in patients aged over 70 years [73]. The incidence of falls was significantly higher in the 60,000 IU dose group (66.9%; 95% CI 54.4 to 77.5%) and the 24 000 IU plus calcifediol group (66.1%; 95% CI 53.5–76.8%) group compared with the low dose (24,000 IU) group (47.9%; 95% CI 35.8–60.3%) (*p* = 0.048). This was despite the fact that the high dose groups were more likely to achieve 25 hydroxy-D levels of > 75 nmol/L.

In order to appropriately interpret the many studies on vitamin D supplementation with varying results, factors that should be taken into consideration include study power, dose used, repeated dosing, supplementation type (vitamin D2 vs D3 vs calcitriol), duration of supplementation, case-mix, primary outcome and the event rate of primary outcome [74].

A further subject of controversy is whether dosing regimens can be standardised or whether bespoke dosing is required. This stems from the multiple factors that influence response to vitamin D supplementation [75], such as baseline vitamin D status, age, body weight, skin pigmentation, diet, physical activity and genetics.

### Supplementation in critical illness—how much, which route, how often and when?

Critically ill patients display large variations in pharmacokinetics due to perturbations in pathophysiology. As a result achieving adequate supplementation of vitamin D in this patient group is challenging.

A few studies in adult patients have demonstrated the ability to achieve adequate biochemical levels of 25 hydroxy-D with supplementation. In a pilot study an enteral ultra-high dose of 540,000 IU of vitamin D3 given once to vitamin D-deficient patients was able to rapidly normalise 25 hydroxy-D levels in 80% of patients in a medical ICU [38].

In a study of 33 critically ill patients with sepsis, Mata-Granados et al. [37] demonstrated that 60,000 IU oral cholecalciferol administered on days 0 and 4 significantly increased 25 hydroxy-D levels.

A study of parenteral administration demonstrated that a single dose of intramuscular cholecalciferol of either 150,000 IU or 300,000 IU corrected vitamin D deficiency in the majority (88%) of critically ill patients [42]. However, correction was not achieved with a single dose in patients with extreme illness severity requiring extracorporeal membrane oxygenation (ECMO) support.

A larger double blind, randomised trial performed at five different ICUs of one tertiary hospital, studied administration of high-dose vitamin D3 compared with placebo; only half of patients treated with vitamin D3 achieved serum 25 hydroxy-D levels higher than 75 nmol/L. The authors suggest that this low response may have been related to critical illness-associated compromised gastrointestinal function and to renal and drug-related compromises of the hepatic cytochrome P450 (CYP450) system that is implicated in 25-hydroxylation of vitamin D [40].

Overall, this suggests that parenteral supplementation and potentially repeated dosing may be required to achieve the supplementation target. Depending on the benefit being sought, for example muscle function and recovery, dosing following the acute phase of critical illness and once stabilisation is achieved may indeed be appropriate.

While the above discussion focuses on supplementation of vitamin D with relatively large doses aiming to achieve pleiotropic effects and the uncertainty surrounding the benefits of this approach, substitution therapy in patients in the ICU should be considered. This is based on their lack of sunlight exposure, minimal intake via dietary sources and the insufficient vitamin D content in parenteral and enteral nutrition delivered. The dose could be based on the Endocrine Society recommendations for at risk individuals (1500–2000 IU/day) [44].

### What would be an appropriate endpoint for a clinical trial of vitamin D supplementation in critical illness given the purported pleiotropic benefits?

Choosing a patient-centred clinically relevant outcome for intensive care trials has been an ongoing challenge. With the heterogeneity of patient populations, studies using a mortality endpoint, although appropriate, require very large sample sizes. Furthermore, this approach might mask potential clinical benefits that are achieved in certain subgroups or on certain organ systems.

For a study on vitamin D supplementation in critical illness, while endpoints pertaining to its pleiotropic immune function such as infectious events may be of great relevance, medium term outcomes such as muscle function and bone health would be similarly relevant.

Defining endpoints for phase II trials in intensive care is similarly challenging. A consensus panel concluded that there are no adequately validated end points. However, the following may be potential phase II end points: hospital-free days to day 90, ICU-free days to day 28, ventilator-free days to day 28, cardiovascular support-free days to day 28 and renal replacement therapy-free days to day 28 [76].

The only robust RCT to date in this area [40] is the single-centre phase II study assessing the effect of oral supplementation in deficient patients on hospital length of stay. Although there was no difference in the primary endpoint, the authors observed lower hospital mortality in the severely vitamin D-deficient subgroup and concluded that this finding should be considered hypothesis generating and required further study.

This group is currently undertaking a randomized parallel group trial (VITDALIZE) which will recruit 2,400 critically ill participants with severe vitamin D deficiency to receive either high dose enteral cholecalciferol or placebo. The primary end-point for this study is 28-day mortality. Several of the challenges discussed above have been considered while designing this trial. Another trial which is not currently recruiting (VIOLET) aims to study the effect of early administration of high-dose vitamin D3 in reducing all-cause mortality in vitamin D deficient patients at high risk for acute respiratory distress syndrome (clinicaltrials.gov).

## Conclusions

The putative pleiotropic benefits of vitamin D supplementation warrant further in-depth exploration with adequately powered multi-centre randomised trials.

This would require reliable assessment of baseline vitamin D status and metabolites with standardised assays. The most effective and safe method of supplementation with respect to preparation, dose, route and frequency of administration are all important considerations. In addition, an appropriate population should be identified who are most likely to benefit from supplementation and stratification of the population or a priori subgroups may need to be considered. Finally, relevant patient-centred primary and secondary endpoints will need to be selected.

## Abbreviations

1,25(OH)_2_D: 1,25 Dihydroxy-vitamin D
25 hydroxy-D: 25 Hydroxy-vitamin D
CI: Confidence interval
CYPs: Cytochrome P450 mixed-function oxidases
DBP: Vitamin D binding protein
ECMO: Extracorporeal membrane oxygenation
ICU: Intensive care unit
IOM: Institute of Medicine
LC-MS/MS: Liquid chromatography mass spectrometry
NYHA: New York Heart Association
OR: Odds ratio
PICU: Paediatric intensive care unit
PTH: Parathyroid hormone
RCT: Randomised controlled trial
RR: Relative risk
RTI: Respiratory tract infection
SRM: Standardised Reference Material
UVB: Ultraviolet B light
VDRs: Vitamin D receptors
